# Supplementary material for: Transcriptome Analysis Reveals Vimentin-Induced Disruption of Cell–Cell Associations Augments Breast Cancer Cell Migration
Source: Cells. 2022 Dec 13;11(24):4035. doi: 10.3390/cells11244035 (PMC9776984; doi:10.3390/cells11244035)
Supplement: Supplementary file 1 [file cells-11-04035-s001.zip › DEGs and enrichment analysis.zip!/DEGs and enrichment analysis/WT_VIMvsVector KEGG/ALL/WT_VIMvsVector_all.dot.pdf]

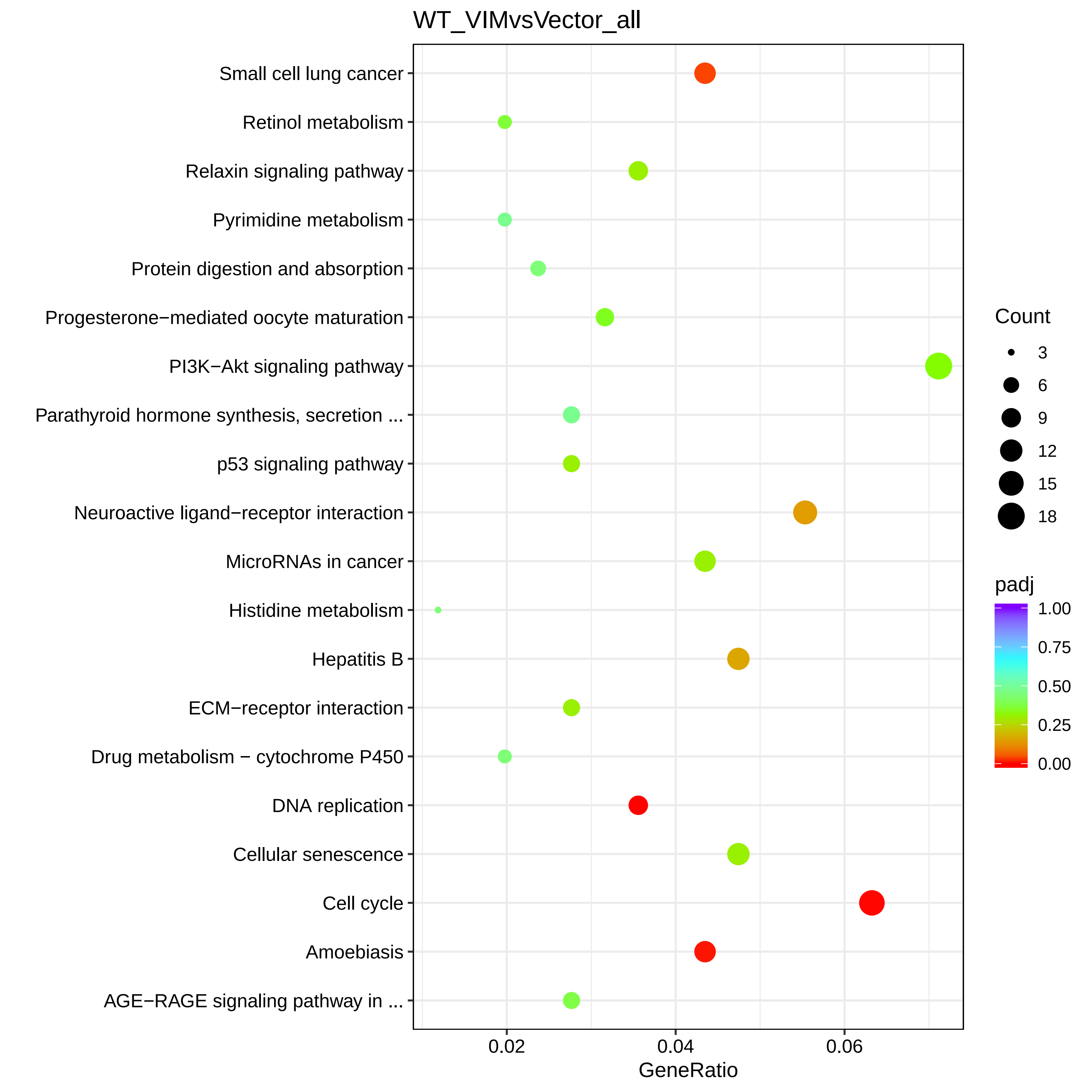
WT_VIMvsVector_all
Small cell lung cancer
Retinol metabolism
Relaxin signaling pathway
Pyrimidine metabolism
Protein digestion and absorption
Progesterone−mediated oocyte maturation
PI3K−Akt signaling pathway
Parathyroid hormone synthesis, secretion ...
p53 signaling pathway
Neuroactive ligand−receptor interaction
MicroRNAs in cancer
Histidine metabolism
Hepatitis B
ECM−receptor interaction
Drug metabolism − cytochrome P450
DNA replication
Cellular senescence
Cell cycle
Amoebiasis
AGE−RAGE signaling pathway in ...
0.02
0.04
0.06
GeneRatio
Count
3
6
9
12
15
18
padj
1.00
0.75
0.50
0.25
0.00
